# Two-photon fluorescence and second harmonic generation characterization of extracellular matrix remodeling in post-injury murine temporomandibular joint osteoarthritis

**DOI:** 10.1371/journal.pone.0214072

**Published:** 2019-03-21

**Authors:** David A. Reed, Mamoru Yotsuya, Polina Gubareva, Peter T. Toth, Andrew Bertagna

**Affiliations:** 1 University of Illinois at Chicago, Department of Oral Biology, Chicago, United States of America; 2 Tokyo Dental College, Department of Fixed Prosthodontics, Tokyo, Japan; 3 University of Illinois at Chicago, Research Resources Center Imaging Core, Chicago, United States of America; University of South Carolina, UNITED STATES

## Abstract

End stage temporomandibular joint osteoarthritis (TMJ-OA) is characterized by fibrillations, fissures, clefts, and erosion of the mandibular condylar cartilage. The goal of this study was to define changes in pericellular and interterritorial delineations of the extracellular matrix (ECM) that occur preceding and concurrent with the development of this end stage degeneration in a murine surgical instability model. Two-photon fluorescence (TPF) and second harmonic generation (SHG) microscopy was used to evaluate TMJ-OA mediated changes in the ECM. We illustrate that TPF/SHG microscopy reconstructs the three-dimensional network of key fibrillar and micro-fibrillar collagens altered during the progression of TMJ-OA. This method not only generates spatially distinct pericellular and interterritorial delineations of the ECM but distinguishes early and end stage TMJ-OA by signal organization, orientation, and composition. Early stage TMJ-OA at 4- and 8-weeks post-injury is characterized by two structurally distinct regions containing dense, large fiber collagens and superficial, small fiber collagens rich in types I, III, and VI collagen oriented along the mesiodistal axis of the condyle. At 8-weeks post-injury, type VI collagen is locally diminished on the central and medial condyle, but the type I/III rich superficial layer is still present. Twelve- and 16-weeks post-injury mandibular cartilage is characteristic of end-stage disease, with hypocellularity and fibrillations, fissures, and clefts in the articular layer that propagate along the mediolateral axis of the MCC. We hypothesize that the localized depletion of interterritorial and pericellular type VI collagen may signify an early marker for the transition from early to end stage TMJ-OA, influence the injury response of the tissue, and underlie patterns of degeneration that follow attritional modes of failure.

## Introduction

Temporomandibular joint osteoarthritis (TMJ-OA) is a clinical syndrome that includes arthralgia, limited joint mobility, and diminished quality of life [1, 2], and is associated with dysfunctional remodeling of the mandibular condylar cartilage (MCC) and degeneration. In early stage TMJ-OA, the fibrous layer of the MCC thickens as the articular surface flattens and expands [3, 4]. This fibrous tissue is similar in composition and appearance to granulation tissue [5, 6]. These changes in the organization, composition, and integrity of the extracellular matrix (ECM) influence the cellular microenvironment of the tissue and promote pathogenic signaling through altering the bioavailability of growth factors [7], cell-matrix interactions [8], and/or material property dependent biophysical stimuli [9]. End stage TMJ-OA is the result of unresolved chronic signaled dysfunction and is characterized by hypocellularity, matrix fibrillations, fissures, clefts, and erosion of the MCC. Critically, end stage TMJ-OA tissue is unresponsive to conservative therapy [10]. Thus, there is a critical need for defining how alterations in pericellular and interterritorial delineations of the ECM distinguish early and end stage TMJ-OA.

Dual two-photon fluorescence (TPF) and second harmonic generation (SHG) microscopy is ideally suited for defining collagen matrix properties in cartilage pathophysiology. TPF is similar in many ways to standard immunofluorescent techniques but can be used together with whole mount immunolabeling to achieve three-dimensional mapping of epitopes within the matrix. SHG is a complementary technique that utilizes the non-linear interaction of incident laser beams with dense non-centrosymmetric media such as fibrillar collagens to generate new photons with twice the energy or exactly half of the wavelength of the incident light [11–13]. This is achieved without exogenous fluorescent markers and without photobleaching or photodamage. Together, SHG and TPF microscopy can reconstruct multiple, distinct matrix components simultaneously, a technique used successfully in several studies to characterize limb cartilage [11, 14–18].

SHG microscopy is ideally suited for characterizing matrix changes occurring in the MCC since it is non-specific to fibrillary collagens [19]. The MCC ECM is composed of primarily fibrillar type I, II, and III collagens. Type I and III collagens are localized to the superficial layer cartilage while type II collagen is localized to the chondroblastic region [19]. The ratio of type I and III collagens is critical in the remodeling phase of dermal wound healing and is a biomarker of tissue fibrosis [20, 21]. Type III collagen is elevated during TMJ dysfunction [22] but the organization and localization of changes in fibrillar collagens has not been satisfactorily resolved at different stages of TMJ-OA progression.

Among the micro-fibrillar collagens, there has been substantial interest in type VI collagen in cartilage pathologies because it is the most abundant molecule of the pericellular matrix surrounding the chondrocyte [23] and is the primary determinant of the material properties of the pericellular matrix [9]. Injury to the MCC may activate chondro-progenitor cells in the superficial layer [24, 25], particularly from the medial and lateral margins. Type VI collagen binding molecules such as neuron/glial antigen 2 have been shown to internalize at the site of injury during the progression of TMJ OA [26]. Type VI collagen is a critical molecule for cell migration and proliferation and may be locally expressed to facilitate the migration of these cells in response to injury [23, 27, 28]. Dysfunctional remodeling may promote type VI collagen synthesis at the site of injury as a mechanism of chondroprotection [9, 23]. The organization and localization of type VI collagen in nascent and established post-injury chondrocytes has not been satisfactorily resolved. SHG microscopy will reconstruct the pericellular matrix of cartilage but is not specific to type VI collagen [11].

Here we utilize dual SHG and immunolabeled type VI collagen TPF microscopy to evaluate the changes in the composition, organization, and orientation of key ECM collagens prior to and during the formation of degenerative changes in the MCC. Further, we use immunofluorescence and picrosirius enhanced polarizing microscopy from serial sections of healthy and diseased TMJs to validate the TPF and SHG reconstructions.

## Results

### Extracellular matrix remodeling during early and end stage TMJ-OA alters the orientation of superficial layer collagens

**Fig 1:**
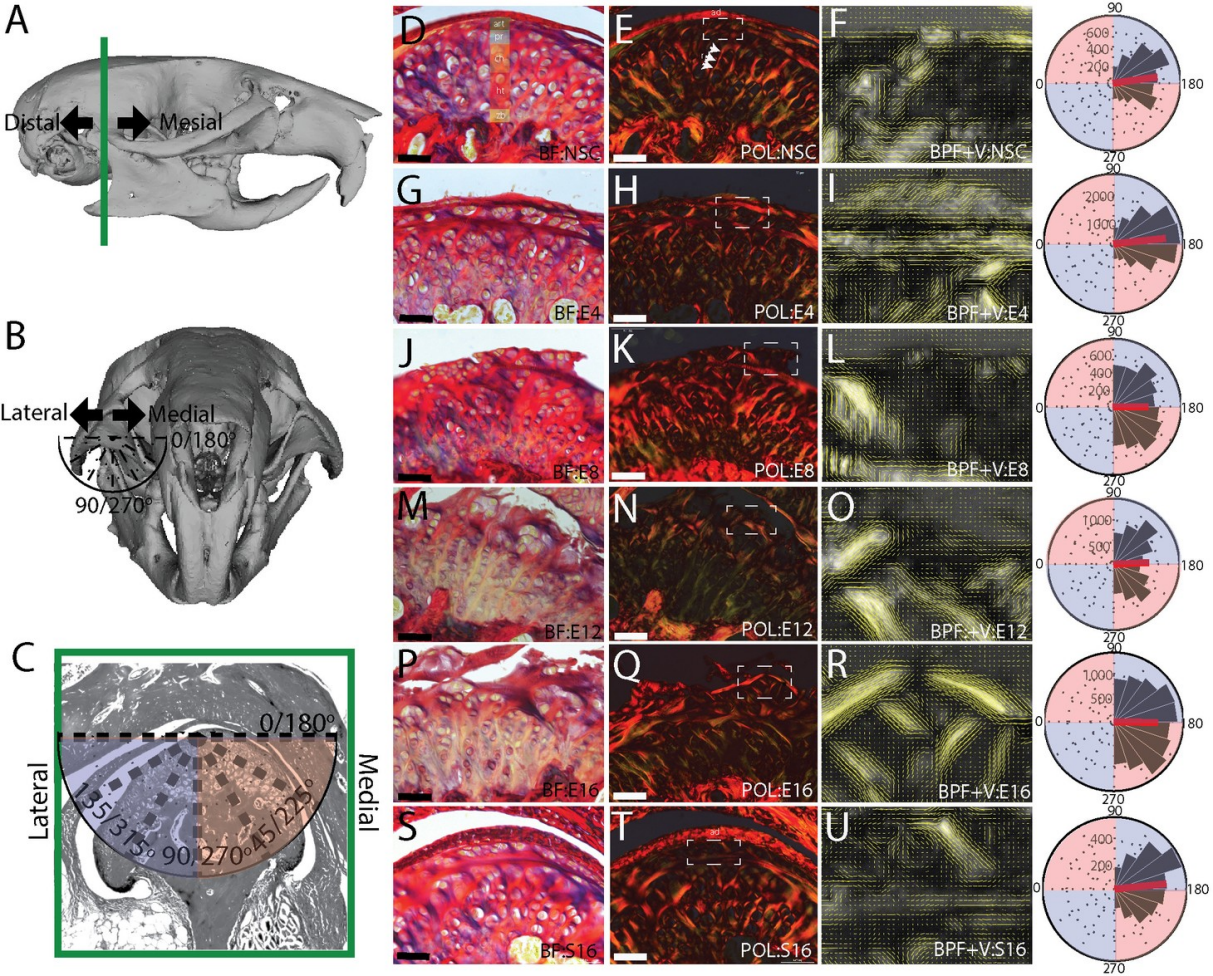
Picrosirius enhanced polarizing microscopy of TMJ-OA. Changes in the organization and orientation of the superficial layer extracellular matrix associated with the progression of TMJ-OA. Reconstructions of a mouse cranium and mandible defining the mesiodistal axis, the mediolateral axis, and orientation of the vectors in the frontal anatomical plane (A-C). Tissue from a non-surgical control (D-F) is compared to 4 (G-I), 8 (J-L), 12 (M-O), and 16 (P-R) week post-injury and 16 week sham controls (S-U). The mandibular cartilage has a heterogenous cell population defined in D: articular (art), proliferative (pr), chondroblastic (ch), hypertrophic (ht) and the zone of bone formation (zb). BF labeled images are picrosirius enhanced bright field, POL labeled images are picrosirius enhanced circular polarizing light microscopy, BPF+V are band pass filtered POL images with vector field mapping of fiber orientation. All images are representative of the experimental group (E8 and S16 n = 3; all others n = 4). Rose plots illustrate the distribution of vector orientations from all samples in each group with vector orientations defined in C. The red line on the rose plot represents the population mean. Vector field is plotted from a quantitative orientation analysis from a region of interest defining the superficial layer cartilage defined in the BF image by a dashed box. All vector lengths are scaled to energy density. Scale bars equal 50 μm and BPF+V image are zoomed to a width of 50 μm. Medial is to the right in all images.

To characterize changes in the organization of the extracellular matrix (ECM) in mandibular condylar cartilage (MCC) during the progression of TMJ-OA, we analyzed the picrosirius red enhanced birefringence of collagen under circular polarizing light. Typical of articular cartilages, the deep MCC is organized into thick bands that form distinct radial and transitional zones [29]. The radial region is characterized by vertically/diagonally orientated collagen bundles that terminate apically as intersecting arches near the superficial layer cartilage and form a thin layer of dense collagens birefringent in a range of colors from weak green to dark red (Fig 1D and 1E). The collagen fibers in the thin superficial layer are oriented primarily along the mediolateral axis, and the collagen fiber bundles in the radial region are oriented approximately 30–45° from the articular surface (Fig 1F). This results in a bimodal distribution of collagen fiber orientations, lacking symmetry about the median orientation (Table 1). At 4-weeks post-injury, the condyle has flattened and the superficial layer has thickened. This thicker superficial layer is composed of collagen fibers oriented mostly in the mediolateral direction (Fig 1G and 1H). The mean fiber orientation is significantly different from the non-surgical control, being more transverse than vertical, with the increase in the overall number of transverse vectors normalizing the vector orientation distribution (Table 1, Fig 1I). At 8-weeks post-injury, the first evidence of cartilage degeneration appears as a disorganization of the superficial layer ECM, localized to the central and medial articular surface (Fig 1J and 1K). The mean collagen fiber orientation is significantly different from the non-surgical control, the variance has increased, and the distribution is no longer normalized about the median (Table 1; Fig 1L). At 12 (Fig 1M and 1N) and 16-weeks (Fig 1P and 1Q) post-injury, the superficial layer has fibrillations, fissuring, clefts, and erosion. These changes in the superficial layer result in fewer transverse collagen fibers at 12-week post-injury (Fig 1O), yielding a bimodal distribution (Table 1). In 16-week sham controls, the overall structure of the MCC appears similar to the non-surgical control and the superficial layer is intact. The vector distributions of the sham and non-surgical controls are both asymmetric about the median, but the mean vector orientation is significantly different (Table 1).

**Table 1:** Circular statistical analysis from polarizing microscopy.

	NSC	E4	E8	E12	E16	S16
Mean	3.01	3.04	3.15	3.09	3.16	3.04
n	4	4	3	4	4	3
ANOVA	* *	*p = 0*.*02*	*p < 0*.*001*	*p < 0*.*001*	*p < 0*.*001*	*p = 0*.*04*
Variance	0.25	0.22	0.35	0.36	0.32	0.26
Symmetry	*p < 0*.*001*	*p = 0*.*07*	*p = 0*.*02*	*p < 0*.*001*	*p = 0*.*14*	*p < 0*.*001*

Circular statistical analysis of quantitative orientation analysis from bandpass filtered picrosirius enhanced polarizing microscopy images. All values reported in radians.

### Dual TPF/SHG reconstructions of the pericellular and interterritorial matrix distinguish early and end stage TMJ-OA

**Fig 2:**
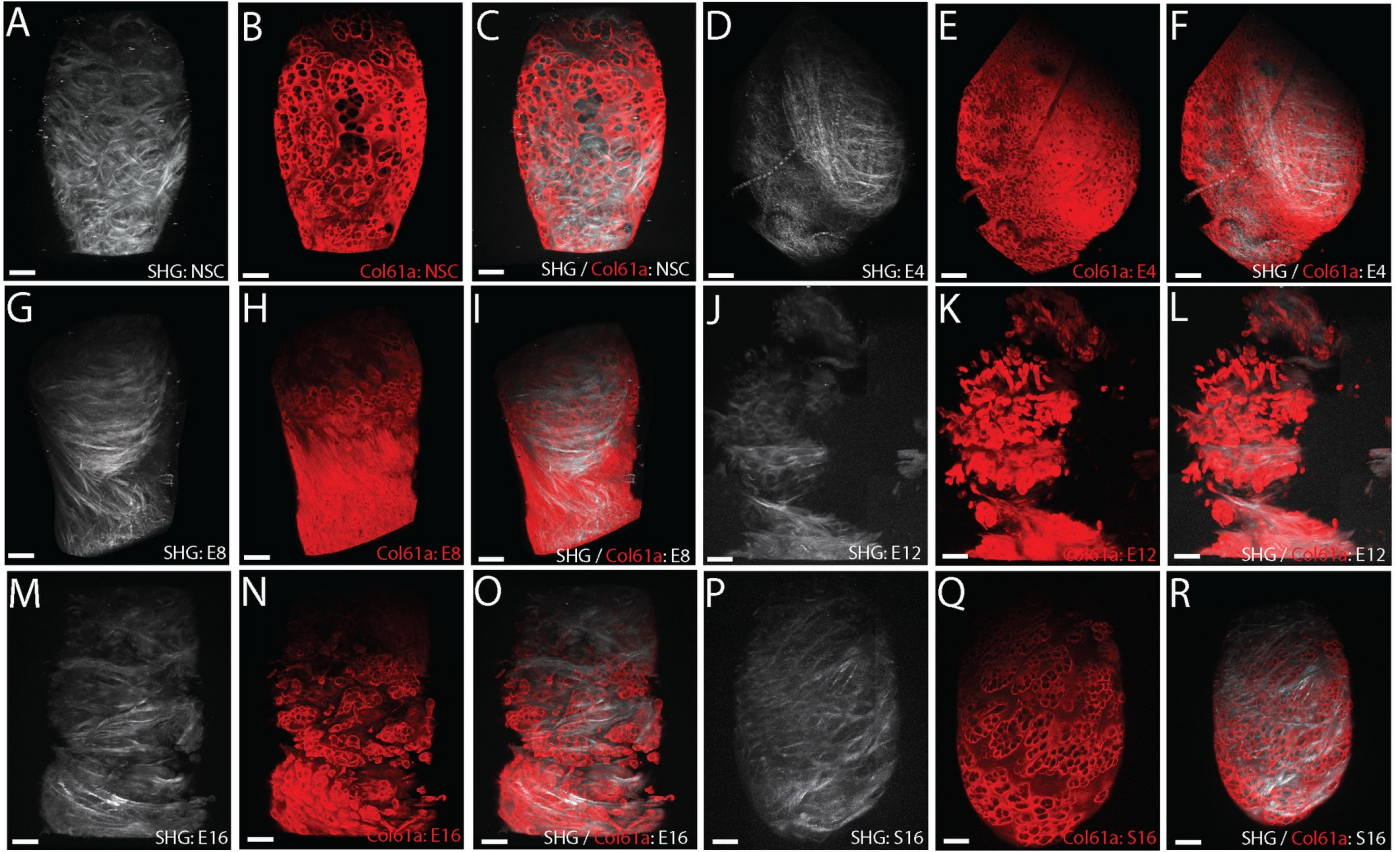
TFP and SHG microscopy of TMJ-OA. Type VI collagen TPF and SHG reconstructions illustrating TMJ-OA associated changes in the organization of both the PCM and the underlying interterritorial ECM in the mandibular condylar cartilage that distinguish early and end stages of the disease. Non-surgical control tissue (A-C) is compared with 4 (D-F), 8 (G-I), 12 (J-L) and 16 (M-O) week post-injury tissue, and 16-week sham controls (P-R). SHG signal reconstructions are represented in white. Whole mount type VI collagen TPF reconstructions are represented in red. All images are superior views from three-dimensional reconstructions of the mandibular condylar cartilage. Scale bars equal 50 μm. Mesial is to the top and medial is to the right in all images. Consistent with the presentation of early stage TMJ-OA, note the presence of thin fiber collagens in the superficial layer cartilage in 4 (D) and 8 (G) week post-injury tissues. Consistent with the presentation of end stage TMJ-OA, note dislocations in the integrity of the SHG and TPF reconstructions in 12 (J-L) and 16 (M-O) week post injury tissues. All reconstructions are representative of experimental groups (E12 and S16 n = 3; all others n = 4).

To further characterize the changes in the ECM preceding and concurrent with the transition from early to end stage TMJ-OA, we imaged healthy and diseased tissues using dual type VI collagen two-photon fluorescence (TPF) and second harmonic generation (SHG) microscopy. The SHG signal reconstructs the three-dimensional organization of fibrillar collagens and is similar in organization to the picrosirius enhanced polarizing microscopy images. SHG positive collagens are arranged into bundles that form intersecting arches near the transitional zone that coalesce into a superficial layer covering the articular surface (Fig 2A). The SHG signal does not label pericellular delineations typical of type VI collagen using our scan settings. Whole mount immunolabeling of type VI collagen imaged using two-photon fluorescence (TPF) does reconstruct this pericellular matrix in the superficial layer ECM (Fig 2B). The general organization of pericellular type VI collagen from TPF matches that observed from confocal immunofluorescence [26, 30], with the strongest stain encapsulating the cells and weak staining in the interterritorial matrix between cells. SHG and superficial layer TPF colocalization is weak in non-surgical control MCC (Fig 2C).

**Fig 3:**
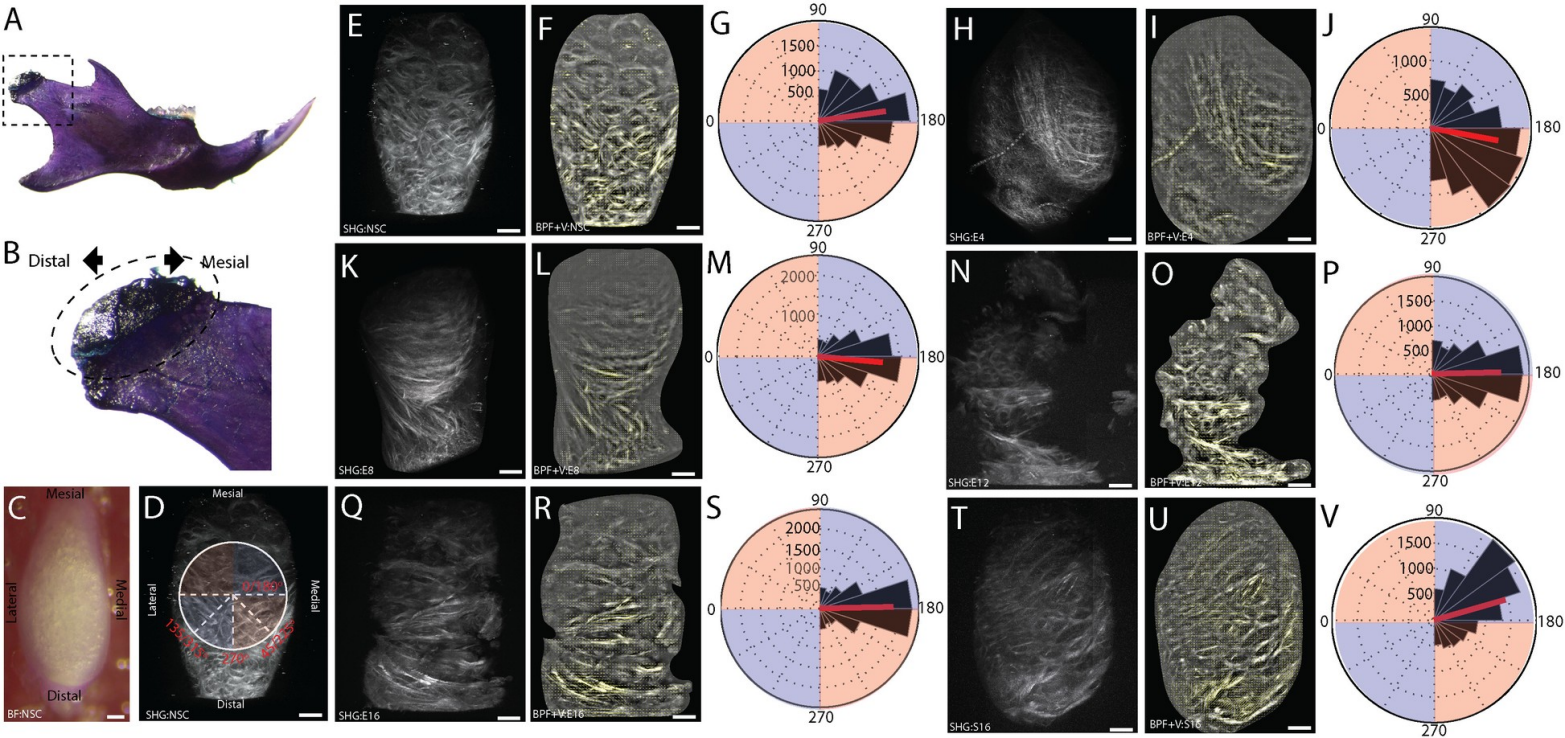
Quantitative orientation analysis of SHG. Quantitative orientation analysis from a band pass filtered SHG reconstructions illustrating TMJ-OA associated changes in the organization of interterritorial ECM in the mandibular condylar cartilage. The orientation of the mandibular condylar cartilage (MCC) is defined (A-B), with a bright field images of the embedded MCC (C) and the SHG scan of that sample defining the mesiodistal axis, the mediolateral axis, and orientation of the vectors. Mesial is to the top and medial is to the right in all images. Non-surgical control tissue (E-G) is compared with 4 (H-J), 8 (K-M), 12 (N-P), and 16 (Q-S) week post-injury and 16-week sham-control (T-V) tissues. All vectors lengths are scaled to energy density. All vector fields are representative of the experimental group (E12 and S16 n = 3; all others n = 4). The vector orientations of each reconstruction are compared using rose plots with the mean orientation plotted in red. 180° represents a mediolateral vector orientation. Scale bars equal 50 μm.

At 4- and 8-weeks post-injury, the SHG/TPF reconstructions illustrate that the extracellular matrix of the superficial layer is composed of mostly small fiber collagens arranged in a similar orientation and there are no signs of fibrillations, fissuring, clefts, or erosion (Fig 2D–2I). The mean orientation of the vector field describing the collagen fibers from 4- and 8-weeks post-injury tissue is significantly different from the non-surgical controls (Fig 3E–3M; Table 2). In the 4-week tissue, the distribution of vector orientations is bimodal and not symmetric about the median orientation (Table 2). At 12- and 16-weeks post-injury, signs of matrix degeneration appear in the superficial layer, with dislocations in TPF/SHG signal intensity that match the structure of matrix fibrillations, fissures, and clefts (Fig 2J–2O). The mean orientation of the vector field is significantly different from non-surgical controls (Table 2). Both the 12 and 16-week distributions are symmetric around the median (Fig 3N–3S; Table 2). Similar to the results of the picrosirius enhanced polarizing data, the mean orientation of the sham control vector field is significantly different from the non-surgical control (Fig 3T–3V; Table 2).

**Table 2:** Circular statistical analysis from SHG quantitative orientation analysis.

	NSC	E4	E8	e12	e16	S16
Mean	3	3.33	3.23	3.12	3.12	2.86
n	4	4	4	3	4	3
ANOVA		*p < 0*.*001*	*p < 0*.*001*	*p < 0*.*001*	*p < 0*.*001*	*p < 0*.*001*
Variance	0.26	0.3	0.22	0.24	0.2	0.26
Symmetry	*p = 0*.*18*	*p < 0*.*001*	*p = 0*.*06*	*p = 0*.*23*	*p = 0*.*56*	*p < 0*.*001*

Circular statistical analysis of the quantitative orientation analysis from bandpass filtered SHG reconstructions. All values reported in radians.

### The ECM of the superficial layer in early stage TMJ-OA is composed of small fibered type I/III collagens oriented along the mesiodistal axis

**Fig 4:**
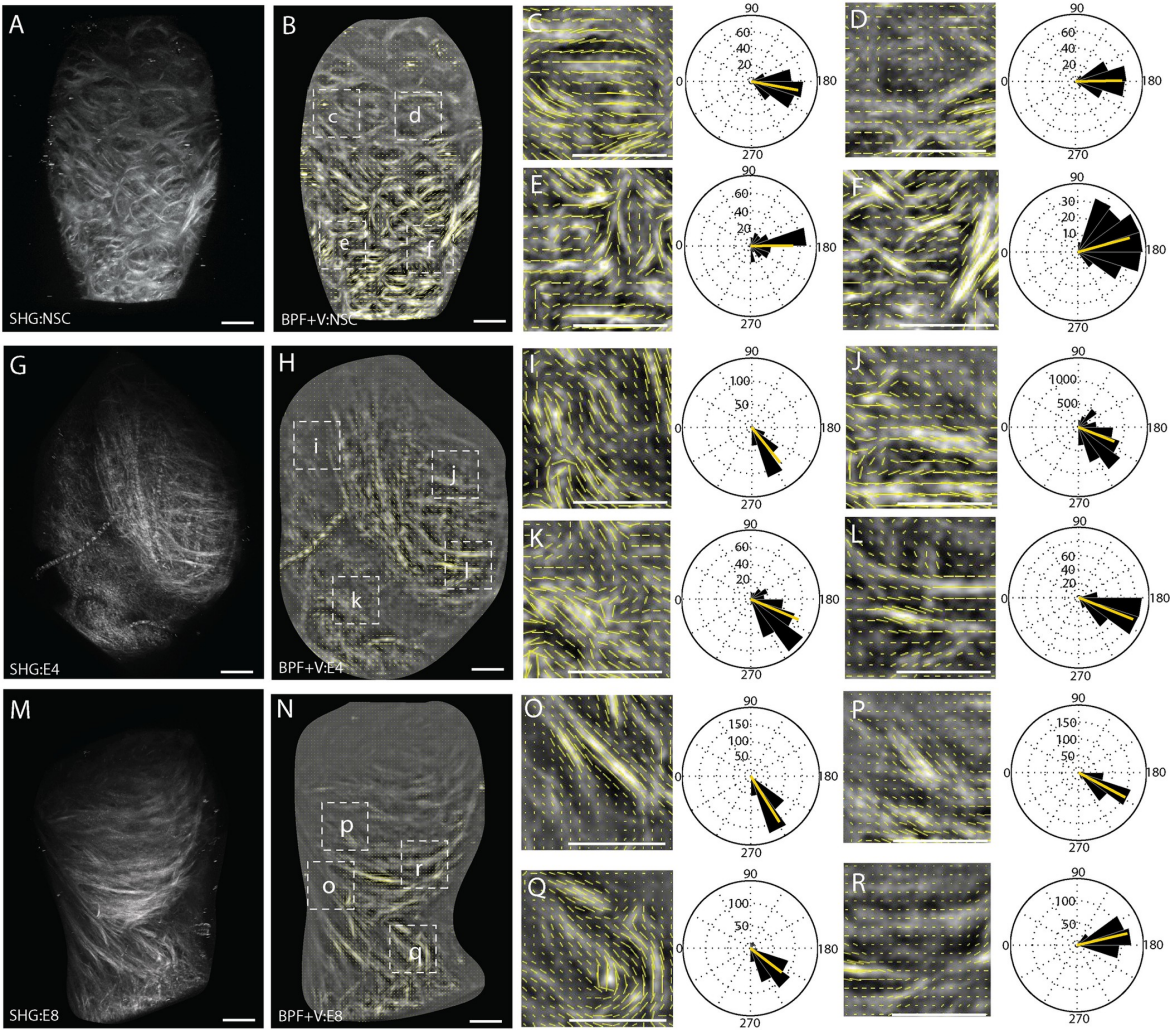
Regional quantitative orientation analysis of early stage TMJ OA. Vector field analysis from a band pass filtered SHG reconstructions illustrating regional variations in the organization of interterritorial ECM in the mandibular condylar cartilage during TMJ-OA. Non-surgical control tissue (A-E) is compared with 4 (F-J) and 8 (K-O) week post-injury tissues. To quantify regional variations in the vector field, four distinct regions of interest are defined from the whole tissue SHG reconstruction as defined in A, F, and K. Cubic spline gradient defined vector field analysis was carried out for each region of interest with vector length scaled to energy density. Scale bars equal 50 μm. Mesial is to the top and medial is to the right in all images. The vector orientations of each reconstruction are compared using rose plots with the mean orientation plotted in red. 180° represents a mediolateral vector orientation.

SHG signal reconstructions of early stage TMJ-OA illustrate that the superficial layer ECM is composed of small fiber collagens that are oriented primarily along the mesiodistal axis of the condyle (Fig 2D and 2G) and have a mean orientation that is significantly different from the non-surgical control (Table 2). Further, the distribution of the vector field describing the 4-week post-injury tissue is bimodal, breaking symmetry about the median. To further evaluate these unique properties of the ECM in early stage TMJ-OA, a quantitative orientation analysis was performed on four distinct regions of the SHG signal reconstruction as a heuristic measure of variance in MCC collagen fiber orientation. In non-surgical controls, the mean vector orientation is transverse along the mediolateral axis of the condyle in all regions sampled (Fig 4A–4E). In the 4 and 8-week post-injury tissues, thin fibered collagens tend to be oriented 30–45° from the mediolateral axis of the condyle (Fig 4G, 4I, 4L and 4N) while thicker bundles tend to be oriented along the mediolateral axis (Fig 4H, 4J, 4M and 4O). This likely accounts for the significant non-symmetry of the vector distribution (Table 2). Circular statistics are excluded from the regional quantitative orientation analysis because of the regions of comparison are non-homologous and varied radially about the circumference of the condyle.

**Fig 5:**
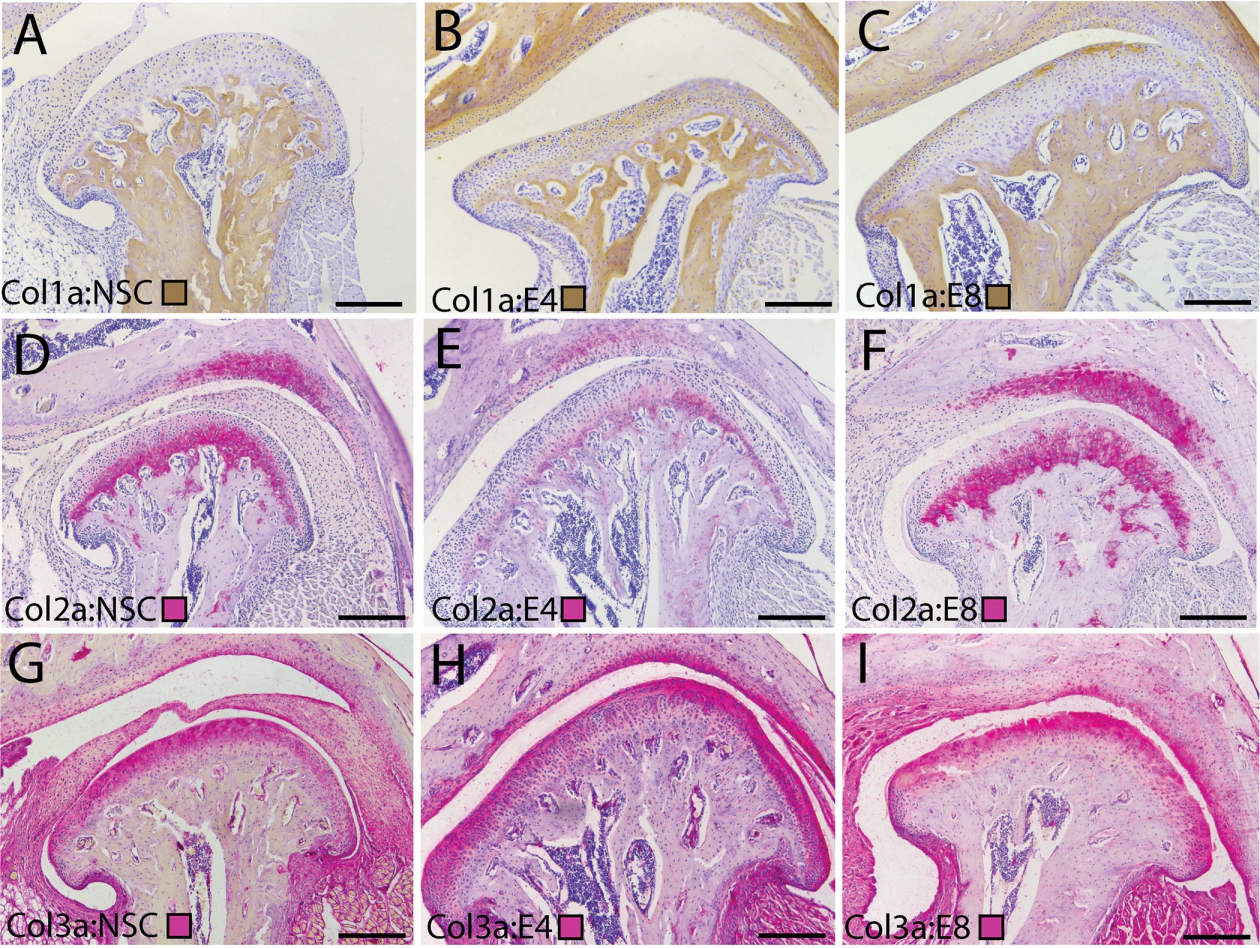
Changes in superficial layer collagens during early stage TMJ OA. Immunolabeling of fibrillar collagens in the TMJ illustrating changes in the composition of the superficial layer extracellular matrix that accompanies the progression of early stage TMJ-OA. Collagen I (A-C) is immunolabeled using a brown chromogen. Collagen II (D-F) and Collagen III (G-I) are immunolabeled using a pink chromogen. Cell nuclei are labeled in purple with hematoxylin. Tissues from non-surgical controls (A, D, G) are compared with 4 (B, E, H) and 8 (C, F, I) week post-injury tissues. All images are representative of their experimental group (n = 3) and taken from homologous regions of the mandibular condyle. Differences in the shape of the condylar reflect variable amounts of condylar flattening, typical of the surgical model. Medial is to the right in all joints. Scale bars equal 50 μm. Note strong staining for Collagens I/III in the superficial layer in post-injury tissues, with Collagen II restricted to the deeper chondroblastic/hypertrophic layers. Primary and secondary antibody controls were negative.

To define the composition the thin fiber SHG signal in early stage TMJ-OA tissues, we immunolabeled spatially homologous sections of the MCC with antibodies against key fibrillar collagens. Type I collagen is found to be substantially elevated in the superficial layer matrix at 4- and 8-weeks post-injury (Fig 5A–5C). Type II collagen is deep to this superficial layer matrix and is localized to chondroblastic layer (Fig 5D–5F). Type III collagen is observed at high levels in the superficial layer matrix in non-surgical control, 4-week, and 8-week tissues (Fig 5G–5I). Together, these data illustrate that the superficial layer of articular mandibular condylar cartilage is dramatically altered during early-stage TMJ-OA, forming thin fibered collagens that are orientated in a similar direction along the mesiodistal axis and composed of types I/III and VI collagen.

### Interterritorial type VI collagen increases in early stage TMJ-OA and then locally degrades in end stage TMJ-OA

**Fig 6:**
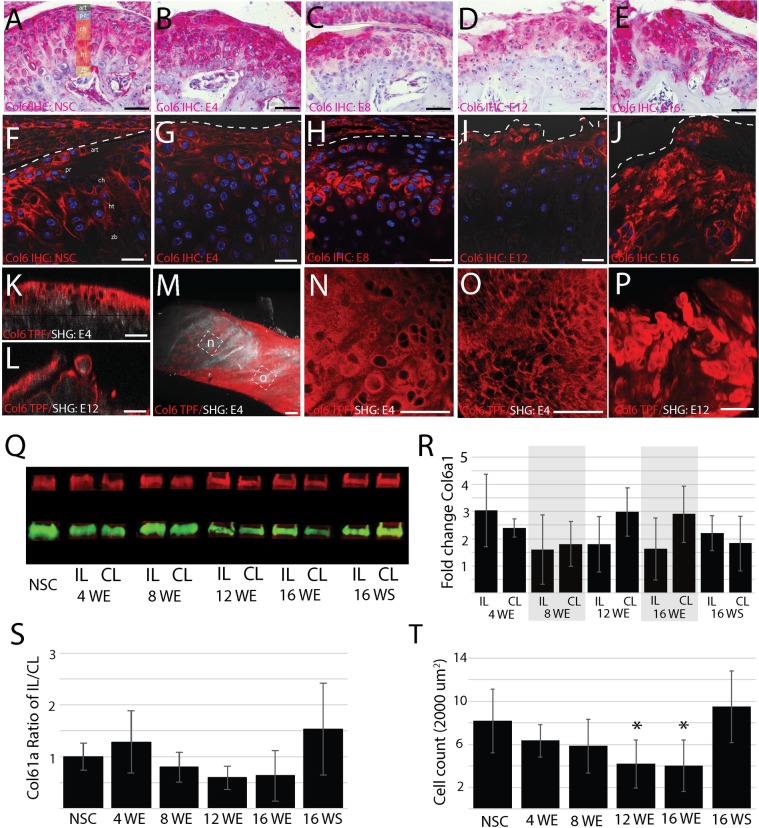
Changes in type VI collagen during TMJ OA. Characterization of changes in the distribution, organization, and level of type VI collagen distinguishing early and late stage TMJ-OA. Chromogenic (A-E) and fluorescent immunohistochemistry (F-J) comparing non-surgical controls (A,F) to 4 (B, G), 8 (C, H), 12 (D, I), and 16 (E, J) week post-injury tissue. Note that the thickened superficial layer immediately below the dotted line in 4-week post-injury tissue is rich in type VI collagen in the interterritorial regions (B, G). The homologous region in 8-week tissue has lower levels of type VI collagen but the superficial layer remains intact (C, H). This superficial layer becomes disorganized, with dislocations and fibrillation in 12 (D, J) and 16 (E, J) week post-injury tissue. Dual type VI collagen TPF and SHG reconstructions illustrate a similar pattern, with high levels of type VI collagen present in the interterritorial matrix of 4-week post-injury tissue (K, M, N) and lose of matrix integrity at 12 weeks (L, P). Note that regional differences in the distribution of type VI collagen are observed in early stage TMJ-OA (M). Large diameter pericellular encapsulation are found near the center of the condyle (N) and small diameter pericellular encapsulation are found near the mesial edge of the condyle. Interterritorial staining is absent from 12-week post-injury tissue (P) Scale bars equal 50 μm. Western blot analysis illustrates that the amount of type VI collagen ipsilateral to injury increases at 4 weeks and then lowers (Q-S). Cell count in the superficial layer also lowers relative to non-surgical controls in late stage TMJ-OA at 12- and 16-weeks post-injury (T).

TPF signal reconstructions illustrate that the organization and expression of type VI collagen can delineate early and end stage TMJ-OA. To further evaluate how type VI collagen changes during the progression of TMJ-OA, we performed immunohistochemistry and western blot analysis. In non-surgical controls, type VI collagen encapsulates articular and proliferative cells in a pericellular matrix. It is expressed at lower levels in the pericellular matrix of chondroblastic and hypertrophic cells. There is some type VI collagen evident in the interterritorial region between cells (Fig 6A and 6F). At 4-weeks post-injury, the thickened superficial layer has high levels of type VI collagen in the interterritorial matrix (Fig 6B and 6G). Ipsilateral to discectomy, the amount of type VI collagen is elevated but not significantly so (Fig 6Q–6S). By 8-weeks post-injury, type VI collagen is mostly absent in the center and medial condyle but strongly expressed in the lateral and medial margins (Fig 2C and 2H). The amount of type VI collagen has returned to the level of the non-surgical control (Fig 6Q–6S). At 12- and 16-weeks post-injury, type VI collagen rich clusters are present in the matrix near the articular surface, the level of type VI collagen contralateral to injury has increased and the level ipsilateral to discectomy has decreased (Fig 6Q–6S). Cellularity in end stage TMJ-OA is also significantly lower (Fig 6T).

The immunohistochemistry and western blot data support the findings from the TPF/SHG reconstructions. Early stage TMJ-OA is characterized by the presence of high levels of interterritorial type VI collagen and small fiber collagens from the SHG signal reconstructions (Fig 6K and 6M). In the region containing the small fiber collagens, the cells are smaller and denser (Fig 6M–6O). In end stage TMJ-OA, the superficial layer is characterized by fibrillations, fissures, and clefts that disrupt the superficial layer. This is evident from the immunohistochemistry analysis (Fig 6D, 6E, 6I and 6J) and the TPF/SHG analysis (Fig 6L and 6P). Together, these data illustrate that the distribution and overall level of type VI collagen changes dramatically in the superficial layer of the mandibular condylar cartilage during the progression of TMJ-OA. Early stage TMJ OA is associated with an increase in type VI collagen associated with small fibered type I/III collagens oriented along the mesiodistal axis of the condyle. The type VI collagen in this remodeled matrix is then locally degraded prior to the formation of fibrillations, fissures, clefts, erosion, and hypocellularity characteristic of end stage TMJ-OA.

## Discussion

Here we illustrate that dysfunctional remodeling of the mandibular condylar cartilage (MCC) produces measurable differences in the organization, orientation, and composition of the superficial layer extracellular matrix (ECM) that delineate early and end stage temporomandibular joint osteoarthritis (TMJ-OA). Early stage TMJ-OA is associated with a thickening of the fibrous, superficial layer matrix, a presentation that has been reported clinically and in other preclinical TMJ injury models [3–5, 31–33]. In our murine model, this thickened superficial layer is composed of small fiber type I/III and VI collagens oriented along the mesiodistal axis of the condyle. Type VI collagen becomes locally diminished on the central and medial condyle prior to both hypocellularity and the formation of fibrillations, fissures, clefts, and erosion typical of end stage TMJ OA.

The formation of a thickened, small fiber collagens over the articular surface of the MCC is consistent with reports of pannus-like (or granulation) tissue in TMJ-OA associated with synovitis [5]. Similar pannus-like tissue has also been reported in limb osteoarthritis [34–36] and is associated with a fibrotic response of superficial layer cells to injury [37, 38]. There is strong support for the hypothesis that TMJ-OA pathophysiology is associated with cells favoring a fibrotic phenotype. MCC progenitor cells express α-smooth muscle actin [39, 40], a marker associated with a fibroblastic phenotype [41]. TGF-β1 upregulates α-smooth muscle actin in fibroblasts and promotes fibrotic matrix production *in vivo* and *in vitro* [42]. TGF-β1 stimulates a fibrotic phenotype in chondrocytes, and TGF-β1 knockout mice have attenuated OA progression [43, 44]. TGF-β1 is elevated in TMJ-OA condyles and believed to play an important role in the progression of TMJ-OA [45]. Taken together, there is strong support for early stage TMJ-OA being associated with a fibrotic superficial layer matrix rich in type I and III collagen. While this remodeling of the superficial layer cartilage is consistent with a reparative tissue response, the resulting sustained, altered biophysical and biochemical signaling may be a key initiating factor in the transition from early to end stage TMJ-OA.

Changes in the biophysical microenvironment of the superficial layer cells are predicted based on changes in the composition, orientation, and organization of the ECM. The superficial layer of the MCC typically resists compressive and tractional forces as the condyle translates over the fossa [46]. Changes in the ratio of type I/III collagen impact the material properties of the extracellular matrix [47]. This may explain why overloaded MCC is associated with a low compliance, high stiffness matrix [48]. *In silico* modeling predicts that the superficial layer promotes interstitial fluid pressurization during loading, lowering the deformation of the cartilage, and lowering the frictional forces on the condyle [49]. These data support the hypothesis that early stage TMJ-OA mediated remodeling of the superficial layer may be a protective stress shielding mechanism to lower compliance and decrease the frictional forces resulting from the removal of the articular disc.

End stage TMJ-OA is associated with degenerative changes in the superficial layer matrix such as fibrillations, fissures, clefts, and erosion of the articular cartilage. Since the thickened superficial layer matrix extends across most of the mediolateral axis of the condyle at 4-weeks post-injury, degenerative changes likely initiate in this type I/III and VI rich layer. Most of these degenerative changes appeared to be oriented along the mediolateral axis of the condyle, orthogonal to the articular plane (Fig 2). Both the picrosirius enhanced polarizing microscopy and the SHG signal reconstructions support this observation. At 12- and 16-weeks post-injury, picrosirius enhanced polarizing microscopy of serial sections in the frontal plane illustrate that degenerative changes result in a significant difference in the mean orientation of the vector field 45–90° from the transverse axis of the condyle and a higher variance when compared to non-surgical controls (Fig 1).

SHG signal reconstructions standardized in the superior view illustrate that these degenerative changes yield collagen fibers oriented primarily along the mediolateral axis (Fig 3) and a decrease in the total variance in the orientation of the vector field (Table 2). It should be noted that this result may reflect some bias from the sampling method. The vector field population is calculated from a local neighborhood of pixels. Each collagen fiber and/or bundle cannot be represented by a single vector. Therefore, the mean orientation and variance reported here is dependent on both the energy of the SHG signal and the user defined sampling grid. Despite the assumptions necessary for this approach, there is strong agreement of the polarizing and SHG methods and support for the observation that cartilage degeneration is associated with a shift in the overall orientation of the extracellular matrix. Thus, the emergence of fibrillations, fissures, and clefts extending along the mediolateral axis of the condyle are associated with both visible and measured differences in the direction of mean collagen orientations in the extracellular matrix. This mechanism of failure is consistent with tractional loading on the matrix.

The transition from early to end stage TMJ OA is also associated with type VI collagen dependent changes in remodeled superficial layer of the MCC. In non-surgical control tissues, type VI collagen strongly delineates the pericellular matrix encapsulating the cells. At 4-weeks post-injury, the thickened superficial layer of the MCC is rich in both types I/III collagen and with type VI collagen present at high levels in the interterritorial regions between cells. Polymorphic progenitor cells migrate from the margins of the MCC in this type VI collagen rich region. Migratory progenitor cells influence cartilage repair in osteoarthritis [50]. Type VI collagen both promotes the proliferation of chondrocytes [28] and progenitor cell infiltration during dermal wound healing [27]. Here we show that remodeling of the superficial layer matrix shares some similarities with the matrix response of dermal wound healing, providing further support for the hypothesis that osteoarthritis displays characteristics of a chronic wound [37, 51]. Under this paradigm, interterritorial type VI collagen in the superficial matrix may be facilitating cell migration and proliferation as part of an injury response. The localized depletion of interterritorial and pericellular type VI collagen may signify an early marker for the transition from early to end stage TMJ-OA attenuating the reparative response from nascent progenitor cells and promoting a loss in the integrity of the extracellular matrix that promotes attritional patterns of failure.

## Materials and methods

### Surgical instability mouse model

TMJ DJD was induced by unilateral partial discectomy [10]. Seven-week post-natal, male c57 BL/6J mice (Jackson Laboratory) were anesthetized with ketamine (100 mg/kg) and xylazine (5 mg/kg). The lateral capsule was exposed and incised taking a posterior approach. The articular disc was excised, the joint was irrigated with sterile saline, and the wound was closed with 5–0 Ethilon suture. Sham control surgeries were identical except the lateral capsule and disc remained intact. Experimental endpoints included four time points: 4, 8, 12, and 16 weeks post-injury. Time points were chosen based on previous descriptions of the mouse model [10, 26]. All experiments using vertebrate animals were approved by the University of Illinois at Chicago Animal Care Committee and performed in accordance with the relevant guidelines and regulations.

### Histology and confocal immunofluorescence

Tissue was embedded and sectioned in the frontal plane at an 8 μm thickness. Sections near chondral lesions were chosen for analysis, generally located caudal of center on the mandibular condyle. To characterize the gross organization of the extracellular matrix (ECM), sections were stained with a 0.5% picrosirius red solution for three hours at 56° C (0.5 g Sirius red F3B in 500 ml picric acid). Chromogen immunolabeling of tissue sections was performed with a multiplex mouse-HRP/rabbit-AP IHC kit following the manufacturer protocol (Enzo, MULTIVIEW Plus). For immunofluorescence, all tissue sections were deparaffinize and pre-treated with Collagenase D (3 mg/ml) and Hyaluronidase (2 mg/ml). Sections were then permeabilized with a methanol wash and a 0.5% (v/v) Triton x100 wash. Antigen retrieval steps included two 30-minute Sodium borohydride (5 mg/ml) washes and a 0.01 M citrate buffer wash at 95° C for 10 minutes (pH 6). Blocking was performed in 5% donkey serum (Sigma D9663) and sections were incubated in primary antibody overnight at 4° C. Primary antibodies include Collagen 6α1 rabbit polyclonal (Fitzgerald, 70R-CR009x), Collagen I goat UNLB (Southern Biotech, 1310–01), Collagen II rabbit polyclonal (Abcam, AB34712), Collagen 3α1 rabbit polyclonal (Invitrogen, PA1-28870). Collagen 6α1 was fluorescently tagged with Alexa Fluor donkey anti-rabbit 568. Nuclei were label with DAPI. Fluorescently labeled sections were imaged using a confocal microscope (Zeiss LSM 710 META). A sample size of three individuals was used for each experimental group. Negative control staining included all of the above steps excluding the addition of the primary antibody. The specificity of collagen antibodies was tested by dual immunofluorescence as part of routine validation.

To calculate cellularity, a region of interest (ROI) containing the superficial layer of the mandibular condylar cartilage was chosen measuring 2000 cm^2^. All DAPI positive cells in the ROI were counted (S1 Table). Means from each experimental group were statistically compared using a one-way ANOVA calculated in MATLAB. A sample size of two individuals was used for each experimental group.

### Whole mount immunofluorescence

Two mandibular condyles ipsilateral to the discectomy were extracted, washed for 15 minutes in 1x PBS, and fixed overnight in 4% PFA. Fixed tissue was washed for 2 hours in 1x PBS, and dehydrated in graded methanol (MEOH) with 10-minute washes at 50%, 70%, 95% and 100% MEOH/dH_2_O. Tissue was washed in Dents Bleach for 24 hours at 37° C (66.7% MEOH, 16.7% DMSO, 16.7% 30% H_2_O_2_), then in 100% MEOH for 1 hour at room temperature, and equilibrated to 1 x PBS with 10 minute graded MEOH washes at 95%, 70%, 50% MEOH/1x PBS. Tissue was permeabilized with 2% Triton, 20% DMSO in 1x PBS for 24 hours at 37° C, and then washed for four days at 37° C on a rocker in blocking solution in 1x PBS adjust to 7.4 pH (0.1M Tris, 0.15M NaCl, and 3% donkey serum (Sigma D9663)). For immunolabeling, the primary Collagen 6α1 rabbit polyclonal antibody (Fitzgerald, 70R-CR009x) was added to a freshly made blocking solution described above at a 1:200 concentration and left to incubate for 10 days at 37° C on a rocker. Antibody solution was exchanged every 2 days. Tissue was then washed three time for 30 minutes in 1% triton in 1x PBS. For secondary fluorescent tagging, Alexa Fluor Donkey anti-Rabbit 568 was added to freshly made blocking solution described above at a 1:200 concentration and left to incubate for 10 days at 37°C on a rocker. Secondary antibody solution was changed every 2 days. Tissue was stored in a 4° C refrigerator in 1x PBS and protected from light until imaging. Negative control staining included all above steps excluding the addition of the primary antibody.

### Two-photon fluorescence and second harmonic generation microscopy

Whole mount tissue, mounted in 1x PBS and placed under a water immersion objective lens (Olympus XLUMPlanFL N 20×/1.00 W), was imaged with a Prairie Technologies Ultima In Vivo Multiphoton Microscopy System. The Coherent Chameleon Ultra II two-photon laser was tuned to 920 nm and the backward scattering second harmonic generation (SHG) signal was acquired with a 460/50 nm bandpass filter. Alexa Fluor 568 was excited at 760 nm and emission signal was collected with a 595/50nm bandpass filter. Prairie View software was used for acquiring three-dimensional data (75 μm z-stacks; n = 4 for each group). Two-photon fluorescence (TPF) and SHG data were reconstructed and superimposed using Imaris Image Analysis Software (Bitplane, Version 7.2).

### Quantification of collagen orientation from polarizing and SHG microscopy

All data was collected using the OrientationJ plugin [52] and collagen orientation measurements follow published methods [53]. All images were first cropped to a region of interest including only the collagens and in a standardized orientation. For polarizing microscopy images, the region of interest was restricted to the superficial layer cartilage since this is where cartilage degeneration was localized. The orientation vector field was generated using a cubic spline gradient structure tensor with a gaussian window of 5 pixels, generating a vector map through the evaluation of a local neighborhood. The local neighborhood is determined by a user defined grid size that was set at 25 pixels. This grid size was chosen to optimize visualization and to be approximately 2.5x the size the average collagen fiber bundle (μ = 9.05 +/- 2.28 pixels). There are biases in this approach such as larger collagen bundles being oversampled. However, the advantage is that changes in the orientation of a single bundle are reflected in the mean orientation. For visualizing the vector field map, vector length was calculated based on energy and scaled to 400%. The mean direction of the circular data, median value of the circular data, and the circular variance were calculated using the circular (directional) statistic toolbox is Matlab [54]. Means and variances were compared using the one-factor ANOVA multi-sample test for equal directional means. Distribution symmetry was calculated using the test for symmetry about a median angle.

### Western blot analysis

Mandibular condyles were extracted under a dissecting microscope and all muscle was removed. Tissue samples were washed in ice cold sterile 1x PBS, flash frozen in liquid nitrogen, and mechanically homogenized with a mortar and pestle. Tissue was then lysed with ice cold matrix extraction buffer (30% glycerol, 187.5 mM Tris, 6% SDS, 100 mM DTT) supplemented with Halt Protease Inhibitor Cocktail (Thermo Fisher) for 3 hours and then boiled for 10 minutes. Lysate Insolubles were removed by centrifugation at 14000 g for 15 minutes at 4° C. Protein concentration was determined by Bradford Assay. The supernatant was incubated with and without Chondroitinase ABC (AMSBio, 100330-1A) for 3 hours at 37° C. Lysates were adjusted to a 1x Protein Sample Loading Buffer (Licor, 928–40004), heated at 100° C 5 minutes, run on a 4–15% sodium dodecyl sulfate polyacrylamide gel (SDS-PAGE), and analyzed by Western Blot with antibodies against Collagen VI (SCBT, clone F-8) and a β-actin monoclonal control (Licor, 926–42210). Protein expression was quantified from band fluorescence and adjusted to the internal control (S2 Table) Mean values compared using a one-way ANOVA in MATLAB (n = 3).

## Supporting information

S1 Table Cell density calculation. Cell density data from each experimental group.
(XLSX)

S2 Table Quantitative western blot fluorescence values. Fluorescence values for quantitative western blot analysis.
(XLSX)
